# Fumigant Toxicity and Oviposition Deterrent Activity of Volatile Constituents from Asari Radix et Rhizoma against *Phthorimaea operculella* (Lepidoptera: Gelechiidae)

**DOI:** 10.1093/jisesa/ieaa133

**Published:** 2020-12-11

**Authors:** Mei Wu, Yan Xiong, Rui Han, Wenxia Dong, Chun Xiao

**Affiliations:** 1 Plant Protection College, Yunnan Agricultural University, Kunming, China; 2 School of Chinese Material Medica, Yunnan University of Chinese Medicine, Kunming, China

**Keywords:** *Phthorimaea operculella*, Asari Radix et Rhizoma, fumigant toxicity, oviposition deterrent

## Abstract

*Phthorimaea operculella* (Zeller) is a worldwide pest of potato. Plant-borne chemicals would be potential alternatives of synthetic chemical fumigants against *P. operculella* in the storage. Asari Radix et Rhizoma is derived from the dry roots and rhizomes of *Asarum heterotropoides* Fr. Schmidt var. *mandshuricum*, *A. sieboldii* Miq. var. *seoulense*, or *A. sieboldii*. In this study, fumigant toxicity and oviposition deterrent of volatile constituents from ARR, δ-3-carene, γ-terpinene, terpinolene, eucarvone, 3,5-dimethoxytoluene, and methyleugenol were tested against *P. operculella*. The preliminary verification of preventive and control effects of eucarvone, 3,5-dimethoxytoluene and methyleugenol on *P. operculella* was carried out by simulating warehouse experiments. The results indicated that the six compounds above had fumigation toxic effects on the adults and eggs of *P. operculella*. Among them, δ-3-carene, γ-terpinene, and terpinolene had weaker fumigation effects than those of eucarvone, 3,5-dimethoxytoluene, and methyleugenol. The LC_50_ values of eucarvone, 3,5-dimethoxytoluene, and methyleugenol against adult *P. operculella* were 1.01, 1.78, 1.51 mg/liter air, respectively. The LC_50_ values against egg *P. operculella* were 1.09, 0.55, 0.30 mg/liter air, respectively. The oviposition deterrent experiment showed that only methyleugenol (at 5 and 1 mg/ml) and eucarvone (5 mg/ml) had a substantial oviposition deterrent effect. The simulated warehouse experiment verified that methyleugenol, eucarvone, and 3,5-dimethoxytoluene protected potatoes from *P. operculella* and demonstrated that methyleugenol had the best preventive and control effects. It was concluded that methyleugenol was the active ingredient with the most potential in the volatiles from ARR on *P. operculella* control and merit further study as botanic fumigant.

*Phthorimaea operculella* (Zeller) (Lepidoptera: Gelechiidae) is a worldwide pest of crop plants in the Solanaceae (Trivedi and Rajagopal 1992, Kwon et al. 2017). It is a major pest of potato and is present in more than 10 provinces in China. Among those provinces, the infestations of *P. operculella* in Yunnan, Guizhou, Sichun, and other southwestern regions are particularly serious and plant damage is increasing yearly (Lu and Cao 1983, Zhu et al. 2003, Xu et al. 2019). *P. operculella* infestations occur in fields and during potato storage. In potato storage facilities, *P. operculella* larvae bore into the potato tuber and discharge dark brown feces outside the borehole. This makes the potato inedible and unmarketable and can cause losses as high as 80%. Thus, it is important to prevent and control *P. operculella* during the storage period (Upamanya et al. 2014, Ibrahim 2015). Fumigation is an effective method for potato pest control during storage. However, most chemical synthetic fumigants have restricted applications because of adverse environmental effects, high mammalian toxicity or reduced efficacy due to pest resistance (Qian et al. 1965, Chandel et al. 2008, Ramadan et al. 2020). Plant volatiles represent potential fumigants with safer characteristics. The secondary metabolites produced by plants constitute the chemical defense system of plants against herbivores. Among them, the volatile components of plants play an essential role. Many countries, especially developing countries, have used aromatic plants for pest control during the storage period of foods (Das 1995, Rajendran and Sriranjini 2008, Regnault-Roger et al. 2012, EL Ghanam, 2017, Campolo et al. 2018).

Chinese medicinal material (CMM) Asari Radix et Rhizoma (ARR) is derived from the dry roots and rhizomes of plants in the family Aristolochiaceae: *Asarum heterotropoides* Fr. Schmidt var. *mandshuricum* (Maxim.) Kitag., *A. sieboldii* Miq. var. *seoulense* Nakai, or *A. sieboldii* Miq. It is a commonly used aromatic CMM with antitussive and analgesic effects, and essential oil was main medicinal chemicals of ARR (CPC 2020). Methyleugenol is the main component and the main pharmacological component of ARR essential oil, which has significant antinociceptive and anti-inflammation effects (Yang et al. 2017). Methyleugenol is a common ingredient of the volatiles released by plants, over 450 species from 80 families have it (Tan and Nishida, 2012). For instance, *Hyssopus officinalis* subsp. *aristatus* (Lamiaceae), *Ocimum sanctum* (Lamiaceae), and *Melaleuca armillaris* (Myrtaceae) have methyleugenol content 22.7, 74.7, and 80.6%, respectively (Venditti et al. 2015, Koroch et al. 2017, Siddique et al. 2017). ARR is not eaten by pests during its storage period, and it has been used to protect other medicinal herbs from pest by storing together with them (e.g., ginseng), its volatiles should have insecticidal properties (Ying et al. 2016). Research on ARR volatiles has involved their effects as insecticides, acaricides, and the prevention and control of mosquitoes and ants (Mo et al. 2003, Wang 2007, Perumalsamy et al. 2009, Dan et al. 2010, Wu et al. 2012, Kim et al. 2016, Wang et al. 2018). ARR powders have demonstrated a fumigation effect on *P. operculella* (Wu et al. 2017). Methyleugenol, 3,5-dimethoxytoluene, eucarvone, δ-3-carene, γ-terpinene, and terpinolene are the main components and (or) the active components of volatiles from ARR (Kim and Park 2008, Perumalsamy et al. 2009, Dan et al. 2010, Kim et al. 2016, Wang 2018). This study evaluated the fumigation effects and oviposition deterrent effect of the above six volatiles from ARR on *P. operculella*. The preliminary verification of their preventive and control effects on *P. operculella* was carried out by simulating warehouse experiments to identify potential plant-derived fumigants for *P. operculella* control on potato during storage.

## Materials and Methods

### Chemicals

ARR is produced mainly in Liaoning province, Jilin province, and Shaanxi province, China. According to the existing research reports, the content of the six compounds in volatile of ARR evaluated in this study are shown in Table 1 (Liu and Liu 2010, Du et al. 2011, Wu 2011, Wang et al. 2014, Wang et al. 2020), and the source of pure compounds are shown in Table 2. Eucarvone was dissolved in acetone and the other five compounds were dissolved in *n*-hexane. All reagents used were of analytical grade.

**Table 1.** The contents of six compounds in volatile of ARR from different geographic origin in China

Compounds	Relative content (%)		
	Liaoning	Jilin	Shaanxi
δ-3-Carene	0–12.9	0–5.0	4.3–10.6
γ-Terpinene	0–0.8	0–0.2	0–0.3
Terpinolene	0.1–2.0	0.2–0.6	0.3–1.1
Eucarvone	0.7–19.2	0.3–11.7	1.4–3.7
3,5-Dimethoxytoluene	0.9–21.6	7.7–18.6	5.8–12.1
Methyleugenol	14.6–61.2	20.2–54.6	5.8–33.2

**Table 2.** The six organic pure compounds evaluated in this study

Compounds	MW^*a*^	VP^*b*^	CAS Registry Number	Source
δ-3-Carene	136.2	1.9	13466-78-9	Acros Organics
γ-Terpinene	136.2	1.1	99-85-4	J&K Chemica
Terpinolene	136.2	1.1	586-62-9	J&K Chemica
Eucarvone	150.2	0.07	503-93-5	Shanghai Yuanye
3,5-Dimethoxytoluene	152.9	0.0375	4179-19-5	Aladdin
Methyleugenol	178.2	0.002	93-15-2	J&K Chemica

^*a*^Molecular weight.

^*b*^Vapor pressure at 25°C.

### Insect

The indoor breeding and housing methods of *P. operculella* were similar to those used by Ma et al. (2013). *P. operculella* adults were confined in a cylindrical container (11 × 10 cm) and fed with water containing 10% honey. The top of the container was closed with nylon gauze (24-mesh). A filter paper was placed on top, which served as an oviposition substrate and facilitated egg collection. After egg hatch, the first instar larvae were inoculated on fresh potatoes (variety Hezuo 88) at a density of 20 larvae per potato (≈130g). The infested potatoes were placed in a mesh cage (35 × 35 × 35 cm^3^) in which sterilized sand was provided. Mature larvae emerged from the potato, entered the sand to pupate and the adults emerged. The breeding room conditions were 27 ± 2°C, 50–70% RH, and a 14:10 (L:D) h photoperiod.

### Fumigant Toxicity Bioassay

The determination of fumigation effect was based on the protocol of Yang et al. (2005) with some modifications. The fumigation experiments of adults and eggs in this study were performed in 296-ml conical flasks with stoppers. A filter paper strip (3 × 1.5 cm^2^) was used as a diffusion carrier for the fumigant and was suspended in the center of the container with a thread. In the fumigation experiments with adult *P. operculella*, the filter paper strip was sandwiched between fine gauze mesh to prevent direct contact with adult *P. operculella*. Based on pretest results, the sample compounds were dissolved and prepared as stock solutions, followed by serial dilution to 5–6 concentrations. A micro-syringe was used to draw 25 µl of the compound solution and drop this on the filter paper strip. Solvent only was added dropwise as a control, followed by volatilization at room temperature for 60 s. The flask openings were tightly closed and sealed with parafilm. The fumigation conditions were 28 ± 0.5°C and 50–70% RH.

### Adult *P. operculella*

To determine the fumigation LC_50_ values of the sample compounds to adult *P. operculella*, 10 pairs of *P. operculella* adults (1 d old) were released in the each of corresponding containers with δ-3-carene (11.03–5.32 mg/liter air), γ-terpinene (8.38–4.79 mg/liter air), terpinolene (5.13–3.57 mg/liter air), eucarvone (1.27– 0.61 mg/liter air), 3,5-dimethoxytoluene (1.93–1.59 mg/liter air), and methyleugenol (1.81–0.87 mg/liter air) for a 24 h fumigation. After the fumigation, the adults were transferred to a clean container for 24 h recovery, followed by recording the number of dead insects. We gently probed immobile moths with a dissecting needle and those not responding were considered to be dead. Each treatment was repeated three times.

### Egg *P. operculella*

Based on preliminary tests, the six compounds were most toxic to 4-d-old *P. operculella* eggs. At this time, eggs have turned black. Thus, the LC_50_ values of the toxicity of fumigation of the sample compounds to the *P. operculella* eggs were determined on 4-d-old eggs. Filter paper containing 30 *P. operculella* eggs was cut and hung in the center of each of the corresponding containers. The treatments were δ-3-carene (6.53–1.68 mg/liter air), γ-terpinene (4.23–1.73 mg/liter air), terpinolene (4.57–1.83 mg/liter air), eucarvone (2.32–0.60 mg/liter air), 3,5-dimethoxytoluene (0.85–0.41 mg/liter air), and methyleugenol (0.89–0.15 mg/liter air) for a 24 h fumigation. After fumigation, the eggs of each treatment group were transferred to a clean container and placed in the breeding room. Three days after the *P. operculella* eggs of the control group had hatching, the number of dead eggs of each treatment group was recorded. Unhatched eggs were considered as dead (Panneerselvam and Murugan 2013). Each treatment was repeated three times.

### Dual-Choice Oviposition Bioassay

This study referred to the methods of oviposition bioassay described by Anfore et al. (2014) and Ma et al. (2016) with some modifications. Healthy potatoes (90 ± 10 g each) of similar size were used for the experiment. The sample compounds were formulated into a series of concentrations (0.2, 1, and 5 mg/ml), and then 200 μl of each of the solutions was applied to filter paper and volatilized at room temperature for 5 min. Each filter paper was placed into a glass bottle (6 × 10 cm) containing a potato. The bottle opening was then covered with double gauze, which as the oviposition site of *P. operculella*. The bottle was tightly covered with a plastic cover (the top surface of the cover, 0.5 cm along the edge, was removed in advance to expose the gauze and allow the scent of the corresponding compound in the bottle to volatilize through the gauze and to fix the gauze as well). Filter paper with solvent only was placed in another glass bottle containing a potato as a control. The excess gauze outside the bottle cover was removed, and the bottle opening was further sealed with parafilm. Two glass flasks were placed diagonally in a rectangular container (dimension: 36 × 28 × 14 cm^3^), followed by released eight pairs of mated adult *P. operculella* (1-d-old), feeding them with water containing 10% honey, and covering the container with nylon gauze (24-mesh). The container was placed in a dark room (26 ± 1°C and 50–70% RH). The number of eggs oviposited on the gauze was counted under a microscope on the third day of the test to calculate the selective oviposition deterrent index (ODI). Each treatment was repeated five times.

### Warehouse Experiment

A warehouse situation was simulated as described by Anfore et al. (2014) with some modifications. The experiment was carried out in a room with the dimensions of (height × width × height = 2.1 × 1.7 × 2.1 m = 7.5 m^3^ and conditions of 26 ± 1°C, 50–70% RH, and a 14:10 (L:D) h photoperiod. Before the experiment, the entire room was cleaned and ventilated for 7 d, and it contained three wooden boxes (32 × 32 × 32 cm) with the top opened. The three wooden boxes were placed approximately 1 m apart in a triangular arrangement. A wire mesh was placed 5 cm away from the bottom of the box. A piece of filter paper was hung in the center and filter papers were placed at the four corners of the wire mesh. Eucarvone, 3,5-dimethoxytoluene, or methyleugenol (1 g) was dropped evenly on the filter papers, and 3 kg of healthy potatoes (90 ± 10 g each potato) were placed on the wire mesh. In another room, three wooden boxes were placed under similar conditions, containing 3 kg healthy potatoes as a control. Twenty-five pairs of mated adult *P. operculella* (1-d-old) were released into the room before sealing the room. The infestation of *P. operculella* on the potatoes was checked 15 d later to calculate the rate of damage in the potatoes.

### Statistical Analysis

Morality data were corrected using Abbott’s formula. Probit regression analysis was used to estimate the LC_50_ and LC_90_ (95% confidence interval). The selective ODI was calculated according to the following formula (Sinthusiri and Soonwera 2014): ODI = amount of eggs laid (treatment - control)/amount of eggs laid (treatment + control). The range of ODI was -0.1–+1.0. An ODI ≤−0.3 represents an oviposition deterrent effect, and an ODI ≥+0.3 represents oviposition attraction. An ODI = -0.3–+0.3 indicated no effect on oviposition. Comparison among multiple groups in the simulated warehouse experiment was performed using one-way analysis of variance (ANOVA) and Tukey’s HSD test. The rate of potato damage was calculated using the following formula (Anfora et al. 2014): Rate of potato damage (%) = Number of potatoes infested by larvae/total number of potatoes × 100. SPSS 17.0 software (IBM SPSS Inc., Chicago, IL, USA) was used for statistical analysis of the data. *P* < 0.05 was considered a significant difference.

## Results

### Fumigant Toxicity Bioassay

The experimental results showed that the six compounds had fumigation toxic effects on the adults and eggs of *P. operculella*. Among them, δ-3-carene, γ-terpinene, and terpinolene had weaker fumigation effects than those of eucarvone, 3,5-dimethoxytoluene, and methyleugenol. The LC_50_ was >4.1 for *P. operculella* adults treated with δ-3-carene, γ-terpinene, and terpinolene and the LC_50_ was <1.8 for *P. operculella* adults treated with eucarvone, 3,5-dimethoxytoluene, and methyleugenol. The LC_50_ was >2.6 for *P. operculella* eggs treated with δ-3-carene, γ-terpinene, and terpinolene and the LC_50_ was <1.1 for the *P. operculella* eggs treated with eucarvone, 3,5-dimethoxytoluene, and methyleugenol. Among eucarvone, 3,5-dimethoxytoluene, and methyleugenol, eucarvone had the strongest fumigation effect on the adults and methyleugenol had the strongest fumigation effect on the eggs.

Different development stages of *P. operculella* had different tolerances to fumigation. Overall, eggs (4 d old) were more sensitive to fumigation than adults. The LC_50_ values of the δ-3-carene treatment on adults and eggs were 7.64 and 3.46 mg/liter air, respectively. The LC_50_ values of the γ-terpinene treatment on adults and eggs were 5.98 and 2.75 mg/liter air, respectively. The LC_50_ values of the terpinolene treatment on adults and eggs were 4.14 and 2.63 mg/liter air, respectively. The LC_50_ values of 3,5-dimethoxytoluene treatment on adults and eggs were 1.78 and 0.55 mg/liter air, respectively. The LC_50_ values of methyleugenol treatment on adults and eggs were 1.52 and 0.30 mg/liter air, respectively. Fumigation toxicity of eucarvone treatment on adults and eggs was similar (LC_50_ values were 1.01 and 1.09, respectively) (Tables 3 and 4).

**Table 3.** Fumigant toxicity of six volatile constituents from Asari Radix et Rhizoma against adult *Phthorimaea operculella* after 24-h exposure

Compound	*n* ^*a*^	Slope (± SE)	Lethal concentrations (mg/liter air)		χ ^2^
			LC_50_ (95% CL)	LC_90_ (95% CL)	
δ-3-Carene	360	7.76 ± 1.41	7.64 (7.00–8.36)	11.18 (9.87–14.17)	0.74
γ-Terpinene	360	11.44 ± 2.02	5.98 (5.60–6.35)	7.74 (7.16–8.92)	1.01
Terpinolene	420	13.80 ± 2.57	4.14 (3.95–4.32)	5.13 (4.82–5.80)	0.66
Eucarvone	360	8.55 ± 1.88	1.01 (0.92–1.11)	1.42 (1.24–1.93)	1.57
3,5-Dimethoxytoluene	360	30.68 ± 5.59	1.78 (1.74–1.82)	1.96 (1.90–2.09)	3.14
Methyleugenol	360	7.98 ± 1.56	1.52 (1.40–1.72)	2.20 (1.89–3.01)	1.21

Lethal concentrations and 95% confidence limits (CL) were estimated using probit regression (SPSS 17.0).

^*a*^The total number of insects used for bioassay.

**Table 4.** Fumigant toxicity of six volatile constituents from Asari Radix et Rhizoma against egg *Phthorimaea operculella* after 24-h exposure

Compound	*n* ^*a*^	Slope (± SE)	Lethal concentrations (mg/liter air)		χ ^2^
			LC_50_ (95% CL)	LC_90_ (95% CL)	
δ-3-Carene	540	6.32 ± 0.85	3.46 (3.14–3.82)	5.51 (4.84–6.75)	1.10
γ-Terpinene	540	5.86 ± 0.78	2.75 (2.51–2.99)	4.55 (4.02–5.51)	3.86
Terpinolene	540	6.50 ± 0.98	2.63 (2.39–2.86)	4.13 (3.67–5.03)	1.16
Eucarvone	540	4.49 ± 0.67	1.09 (0.96–1.24)	2.10 (1.76−2.82)	1.64
3,5-Dimethoxytoluene	540	9.48 ± 1.33	0.55 (0.51–0.58)	0.74 (0.69–0.85)	0.84
Methyleugenol	540	3.80 ± 0.53	0.30 (0.26–0.35)	0.66 (0.54–0.90)	4.33

Lethal concentrations and 95% confidence limits (CL) were estimated using probit regression (SPSS 17.0).

^*a*^The total number of insects used for bioassay.

### Dual-Choice Oviposition Bioassay

Within the range of tested concentrations (0.2, 1, and 5 mg/ml), only eucarvone and methyleugenol showed oviposition deterrent effects. Methyleugenol had the strongest effect, and the oviposition deterrent effect decreased with the decrease in methyleugenol concentrations. Both high- (5 mg/ml) and moderate (1 mg/ml)-concentration methyleugenol treatments showed an oviposition different effect with the ODIs of −0.97 and −0.51, respectively. The ODI of the low-concentration (0.2 mg/ml) methyleugenol treatment had an ODI of −0.12. The ODI of high-concentration (5 mg/ml) eucarvone treatment was −0.49, and the ODIs of the moderate- (1 mg/ml) and low-concentration (0.2 mg/ml) eucarvone treatments were 0.15 and 0.01, respectively (Table 5).

**Table 5.** The dual-choice oviposition deterrent of six volatile constituents from Asari Radix et Rhizoma on *Phthorimaea operculella*

Compound	Concentration	Number of eggs laid on the gauze		ODI (±SE)^*a*^	
	(mg/ml)	Treatment	Control	(*n* = 5)	
δ-3-Carene	0.2	189.2	196.2	0.00 ± 0.11	N
	1	164.8	172.4	−0.02 ± 0.10	N
	5	133.6	150.0	−0.04 ± 0.12	N
γ-Terpinene	0.2	114.8	106	0.02 ± 0.12	N
	1	98.4	129.2	−0.15 ± 0.09	N
	5	103.8	151.0	−0.19 ± 0.08	N
Terpinolene	0.2	155.0	158.0	0.00 ± 0.07	N
	1	156.8	161.8	−0.03 ± 0.08	N
	5	111.2	188.2	−0.27 ± 0.06	N
Eucarvone	0.2	125.4	122.8	0.01 ± 0.05	N
	1	144.2	107.0	0.15 ± 0.05	N
	5	69.2	205.2	−0.49 ± 0.06	R
3,5-Dimethoxytoluene	0.2	126.0	118.0	0.04 ± 0.12	N
	1	120.8	135.0	−0.06 ± 0.08	N
	5	149.0	144.6	0.03 ± 0.11	N
Methyleugenol	0.2	164.8	211.0	−0.12 ± 0.04	N
	1	72.6	217.6	−0.51 ± 0.06	R
	5	5	249.6	−0.97 ± 0.03	R

^*a*^ODI oviposition deterrent index, which ranges from −1.00 to +1.00; ODI = −0.3 and below indicated the test solution as repellents (R), and ODI between +0.3 and −0.3 were indicated as neutral response (N).

### Warehouse Experiment

**Fig. 1.**
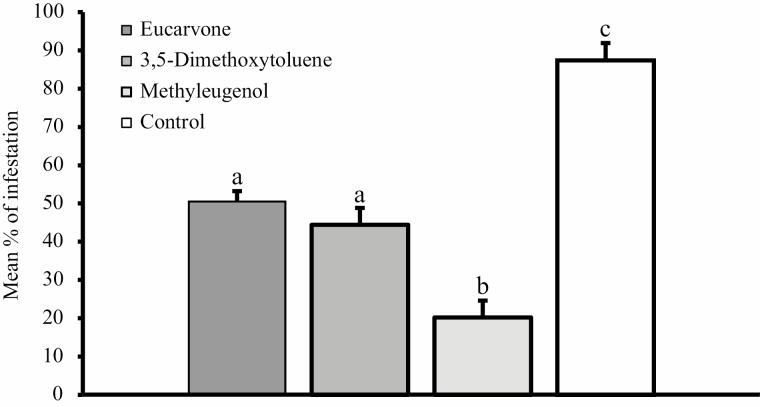
Mean percentage (±SE) of potato tubers damaged by *Phthorimaea operculella* in storage conditions. Different letters on the bars indicate signiﬁcant differences (ANOVA, Tukey’s test: *F* = 42.9; df = 3,11; *P* < 0.001).

In the simulated warehouse experiment, almost all potatoes in the control group were infested by *P. operculella* (rate of potato damage: 87.4%). The eucarvone, 3,5-dimethoxytoluene, and methyleugenol treatment significantly reduced the potato damage (*F* = 42.9; df = 3,11; *P* < 0.001). The rates of potato damage after the eucarvone and 3,5-dimethoxytoluene treatments were not significantly different at 44.4 and 50.5%, respectively. The rate of potato damage after the methyleugenol treatment was 20.2% and this was significantly lower than the eucarvone and 3,5-dimethoxytoluene treatments (Fig. 1).

## Discussion

ARR has a strong aroma, and its volatile oil content is ≥2% (CPC 2020). The components in the volatile oil are complex. Du et al. (2011) isolated 52 components from the volatile oil of ARR. Wang et al. (2018) analyzed the main components of the volatile oil of ARR and showed that phenylpropenes (54.21%) and monoterpenes (24.02%) were the main components. Among the volatiles, the levels of methyleugenol, safrole, and 3,5-dimethoxytoluene were the highest. Methyl eugenol and safrole belong to the phenylpropenes. ARR has a long history of medicinal use, and its volatile oil is the main active ingredient. ARR has toxic to humans, so its dosage should be limited in clinical practice to no more than 1–3 g/d (CPC 2020). The toxicity of ARR mainly comes from the safrole in its volatile oil. Safrole is carcinogenic (Ping et al. 2012, Wang et al. 2018). Although safrole is a component with strong insecticidal and acaricidal activities in the essential oil of ARR, its mammalian toxicity may limit its further development and application.

Aromatic plants with insecticidal effects are sources of screening potential active compounds as plant-derived fumigants. Such as 3,5-dimethoxytoluene, which is the main ingredient of rose aroma and is safe for humans and the environment. The 3,5-dimethoxytoluene is commonly used in the perfume industry and in aromatherapy (Scalliet et al. 2002, Api et al. 2019). Because of the plant protection field research on ARR, 3,5-dimethoxytoluene as an insecticide and acaricide were evaluated. Kim et al. (2016) showed that 3,5-dimethoxytoluene had a fumigation effect on mites. Wang et al. (2018) evaluated the fumigation effect of 3,5-dimethoxytoluene, methyl eugenol, and safrole on *Lasioderma serricorne* and *Liposcelis bostrychophila* and showed that 3,5-dimethoxytoluene had the strongest fumigation effect among the three compounds.

This study evaluated the preventive and control effects of six components in ARR volatiles against *P. operculella.* The fumigation test on adults and eggs of *P. operculella* showed that the fumigation effects of methyl eugenol, eucarvone, and 3,5-dimethoxytoluene were stronger than the effects of δ-3-carene, γ-terpinene, and terpinolene. The oviposition deterrent experiment showed that only methyleugenol (at 5 and 1 mg/ml) and eucarvone (5 mg/ml) had a substantial oviposition deterrent effect. The simulated warehouse experiment verified that methyleugenol, eucarvone, and 3,5-dimethoxytoluene protected potatoes from *P. operculella* and demonstrated that methyleugenol had the best preventive and control effects. Other studies on insecticide, acaricide, and anti-microorganism have confirmed that methyleugenol is the main active ingredient in the volatiles from ARR (Dan et al. 2010, Wu et al. 2012, Yi et al. 2015, Kim et al. 2016). Results of this study indicated that methyleugenol was the active ingredient with the most potential in the volatiles from ARR for the prevention and control of *P. operculella.* Further studies on its mode of action, residues, and influence on potato germination are needed. In addition to screening for the active ingredients in ARR volatiles, studies on the nonvolatile ingredients of ARR should be conducted in the development of ARR in the search for plant-derived pesticides. (−)-asarinin and pellitorine were active components with acaricidal and (or) insecticidal activities, which had been screened from the nonvolatile components of ARR (Perumalsamy et al. 2010, Han 2011).
